# ESBL-Producing *Salmonella enterica* Serovar Typhi in Traveler Returning from Guatemala to Spain

**DOI:** 10.3201/eid2011.140525

**Published:** 2014-11

**Authors:** Juan José González-López, Nuria Piedra-Carrasco, Fernando Salvador, Virginia Rodríguez, Adrián Sánchez-Montalvá, Anna M. Planes, Israel Molina, M. Nieves Larrosa

**Affiliations:** Hospital Vall d’Hebron, Barcelona, Spain (J.J. González-López, N. Piedra-Carrasco, F. Salvador, V. Rodríguez, A. Sánchez-Montalvá, A.M. Planes, I. Molina, M.N. Larrosa);; Universitat Autònoma de Barcelona, Barcelona (J.J. González-López, N. Piedra-Carrasco, M.N. Larrosa)

**Keywords:** Salmonella enterica, Typhi, typhoid fever, ESBL, CTX-M-15, CTX-M, salmonellae, extended-spectrum β-lactamase, β-lactams, bacteria, Americas, Guatemala, Spain, travel, Salmonella enterica serovar Typhi

## Abstract

We report a case of typhoid fever in a traveler returning to Spain from Guatemala that was caused by *Salmonella enterica* serovar Typhi which produced an extended-spectrum β-lactamase (ESBL). This finding demonstrates the presence of ESBL-producing *S. enterica* ser. Typhi strains in the Americas. Enhanced surveillance is necessary to prevent further spread.

*Salmonella enterica* serovar Typhi is the causative agent of typhoid fever, an enteric bacterial infection that results in systemic febrile illness. *S. enterica* ser. Typhi is strictly adapted to humans; its transmission occurs through the fecal-oral route, person-to-person contact, or contaminated water or food. An estimated 22 million new cases of typhoid fever occur each year worldwide, resulting in 200,000 deaths (*1*). *S. enterica* ser. Typhi infection is uncommon in industrialized countries, where infections occur sporadically and mainly in travelers returning from disease-endemic areas and in newly arrived immigrants.

Chloramphenicol was used successfully as the first-line agent for the treatment of typhoid fever from the 1950s through the 1970s. Strains resistant to this compound emerged in 1972, associated with self-transferable *Inc*HI plasmids. Trimethoprim-sulfamethoxazole and ampicillin were then employed, but strains resistant to all 3 drugs arose rapidly during the 1980s and 1990s in southern and Southeast Asia, the Middle East, and Africa (*2**,**3*). To overcome these new resistances, fluoroquinolones were proposed as the drug of choice. However, during the past decade, ciprofloxacin-resistant strains have been reported in the Asian subcontinent (*2*).This situation has resulted in the use of ceftriaxone or cefotaxime as alternatives for treatment of enteric fever (*3*).

Until now, extended-spectrum β-lactamase (ESBL)–producing *S. enterica* ser. Typhi strains have been uncommon and have been described only in a few patients of Asian origin and in travelers returning from that region (*4**–**7*). We report the detection and molecular characterization of an ESBL-producing *S. enterica* ser. Typhi strain isolated in Barcelona, Spain, from a patient with typhoid fever who had traveled to Guatemala.

## The Study

In September 2013, a 41-year-old man residing in Barcelona visited the emergency room of the Vall d’Hebron Hospital, reporting 10 days of fever and pain in the right upper abdominal quadrant, preceded by 3 days of diarrhea. At the time of his hospital visit, the patient had been back in Spain for 4 days after a 5-month stay in rural Guatemala. Physical examination revealed a temperature of 37.8°C and unremarkable blood pressure and heart rate results. Abdominal examination showed tenderness of the right upper and lower quadrants. General biochemistry values and blood cell counts were within reference ranges, but C-reactive protein level and erythrocyte sedimentation rate were elevated. Results of initial blood and stool culture testing were negative. Because of the patient’s persistent abdominal pain and newly documented fever, computed tomographic scan of the abdomen was performed; results showed thickening of the terminal ileum, adjacent fat tissue stranding, and regional lymphadenopathy. Antimicrobial drug treatment with amoxicillin-clavulanate acid was begun. New blood cultures, performed because of the patient’s persistent fever, yielded *S. enterica* ser. Typhi, and the strain produced an ESBL. Subsequently, intravenous ertapenem (1 g/d) was administered for 14 days; the patient experienced complete clinical recovery, and subsequent blood and stool culture results were negative.

The isolate was identified by using the VITEK2 system (bioMérieux, Marcy l’Etoile, France). Serotyping by slide agglutination by using commercial antisera according to the Kauffmann-White scheme yielded the antigenic formula 9,12,[Vi]:d:-. Multilocus sequence typing (MLST), which was done using primers described elsewhere (*8*) with allele sequences and allelic profiles verified in the MLST database (http://mlst.ucc.ie/mlst/dbs/Senterica), showed that the strain belonged to sequence type (ST) 2. This ST is one of the most prevalent among *S. enterica* ser. Typhi; isolates of this ST have been detected in Asia, Africa, and South America (*9*).

Antimicrobial susceptibility to β-lactams was assessed by disk diffusion following Clinical Laboratory Standards Institute recommendations (http://www.clsi.org). Suggestive evidence of ESBL production was observed as synergy between amoxicillin/clavulanate and >1 of the following: cefotaxime, ceftazidime, aztreonam, and cefepime. In addition, MICs of β-lactams, quinolones, trimethoprim/sulfamethoxazole, chloramphenicol, and azithromycin were determined by E-test (bioMérieux) (Table). According to Clinical Laboratory Standards Institute interpretative criteria, the isolate was resistant to all β-lactams evaluated except amoxicillin/clavulanate, piperacillin/tazobactam, cefoxitin, and carbapenems and was susceptible to chloramphenicol, trimethoprim/sulfamethoxazole, nalidixic acid, ciprofloxacin, and gentamicin. The MIC of azithromycin was 4 µg/mL; according to the European Committee on Antimicrobial Susceptibility Testing (http://www.eucast.org), isolates with an MIC ≤16 μg/mL of this drug should be considered wild-type organisms that are expected to respond to treatment (*10*).

**Table:** Susceptibility profiles of the CTX-M-15–producing *Salmonella enterica* serovar Typhi strain from a patient in Spain who had traveled to Guatemala in 2013, compared with profiles of the *Escherichia coli* recipient strain and the transconjugant strain

Antimicrobial agent(s)	MIC, µg/mL		
	*S. enterica* ser. Typhi 301812 (donor)	*E. coli* HB101-Nal (recipient)	*E. coli* HB101-Nal TC-301812 (transconjugant)
Ampicillin	>256	1.5	>256
Amoxicillin-clavulanate	16	3	16
Piperacillin-tazobactam	3	1	1.5
Cefoxitin	12	3	3
Ceftazidime	>256	0.064	8
Cefotaxime	>256	0.016	>256
Cefotaxime-clavulanate	0.19	0.016	0.016
Cefepime	>256	0.032	24
Aztreonam	>256	<0.016	32
Imipenem	0.25	0.38	0.38
Meropenem	0.064	0.023	0.023
Ertapenem	0.064	0.002	0.002
Chloramphenicol	2	2	2
Trimethoprim-sulfamethoxazole	0.047	0.25	0.25
Nalidixic acid	3	>256	>256
Ciprofloxacin	0.023	0.125	0.125
Azithromycin	4	3	3
Gentamicin	1.5	0.75	3
Tobramycin	24	1	>256
Amikacin	32	2	>256

To screen for TEM and SHV β-lactamases, CTX-M ESBL genes, and genes encoding resistance to quinolones (*qnrA*, *qnrB*, *qnrS, qepA, oqxAB,* and *acc(6′)-Ib-cr*), we used PCR as described previously (*11**,**12*). Mutations in *aac(6′)-Ib* that confer quinolone resistance and in the quinolone resistance–determining regions of *gyrA, gyrB*, *parC*, and *parE*, were studied by sequencing (*12**,**13*). These experiments showed that the *S. enterica* ser. Typhi isolate possessed *bla*_TEM-1_, *bla*_CTX-M-15_, and *acc(6′)-Ib* but none of the studied quinolone resistance genes or detectable quinolone resistance–determining region mutations.

IS*Ecp1*, IS*26*, and *orf*477 are elements that previously have been identified in the genetic environment surrounding *bla*_CTX-M-15_. The presence of such elements was investigated by PCR mapping and sequencing in combination with *bla*_CTX-M-15_–specific primers, as reported previously (*14*).These results showed that the ESBL gene was situated between a nontruncated IS*Ecp1* and *orf*477, as noted previously in other *Enterobacteriaceae*.

Identification and characterization of the location of *bla*_CTX-M-15_ was carried out by conjugation, using a nalidixic acid–resistant derivative of *Escherichia coli* HB101 as recipient, PFGE of total DNA of donor and transconjugant digested with S1-nuclease, Southern hybridization with specific probes, and PCR-based replicon typing, as described previously (*14*). These studies showed that the *S. enterica* ser. Typhi *bla*_CTX-M-15_ was located in an IncL/M self-transferrable plasmid of 65 kb that also carried the *bla*_TEM-1_ and *acc(6′)-Ib* genes. MICs to antimicrobial agents for donor, recipient, and transconjugant are shown in the Table.

## Conclusions

We describe an ESBL-producing *S. enterica* ser. Typhi strain isolated from a man in Spain who had traveled to Guatemala. It is well documented that ESBL*-*producing *Enterobacteriaceae* are spreading worldwide. CTX-M-15 is one of the most commonly identified ESBLs; its high prevalence has been driven mainly by the pandemic spread and expansion of the ST131 *E. coli* clonal group. Extended-spectrum cephalosporin resistance has increased during the past few years in nontyphoidal *S. enterica*, principally in developing countries, where such resistance appears to be endemic in some areas (*15*).

To our knowledge, 5 cases of *S. enterica* ser. Typhi resistant to β-lactams by ESBL production have been reported, all with Asian origins (Bangladesh, the Philippines, Iraq, and India) (*4**–**7*). For the isolate originating in the Philippines, SHV-12 was the enzyme responsible for ESBL resistance (*7*); for the other 4 isolates, a CTX-M enzyme was detected, and in 3 of those, the CTX-M-15 variant was identified (*4**–**6*). In our case, however, we found a different genetic environment of *bla*_CTX-M-15_. Specifically, we found a nontruncated IS*Ecp1* upstream of *bla*_CTX-M-15_, whereas in the previously reported Iraq-origin strain, IS*Ecp1* was truncated by IS*26* (*5*). Genetic environments of *bla*_CTX-M-15_–producing *S. enterica* ser. Typhi isolates from India are not clear, but according to the methods used by the authors (*4*), a truncated IS*Ecp1* or a different structure may have been involved. Our results also confirm that the plasmid harboring *bla*_CTX-M-15_, which also carries *bla*_TEM-1_ and *acc(6′)-Ib*, carries an IncL/M replicon.

In summary, we report a case of typhoid fever caused by an ESBL-producing *S. enterica* ser. Typhi isolate from a traveler returning to Spain from Guatemala. This case represents the acquisition of an ESBL-producing *S. enterica* ser. Typhi strains in the Americas. Because typhoid fever is a serious public health issue, meticulous microbiological and epidemiologic investigation of strains of this sort are necessary to prevent further spread of this disease.
